# N-*p*-coumaroyloctopamine ameliorates hepatic glucose metabolism and oxidative stress involved in a PI3K/AKT/GSK3β pathway

**DOI:** 10.3389/fphar.2024.1396641

**Published:** 2024-04-25

**Authors:** Yuechang Huang, Xingmin Zhang, Qian Li, Wende Zheng, Panpan Wu, Rihui Wu, Wen-Hua Chen, Chen Li

**Affiliations:** ^1^ School of Pharmacy and Food Engineering, Wuyi University, Jiangmen, China; ^2^ International Healthcare Innovation Institute (Jiangmen), Jiangmen, China; ^3^ Guangdong Provincial Key Laboratory of Large Animal Models for Biomedicine, Wuyi University, Jiangmen, China

**Keywords:** N-p-coumaroyloctopamine, diabetes, oxidative stress, hyperglycemia, mechanism of action

## Abstract

Type 2 diabetes mellitus is regarded as a chronic metabolic disease characterized by hyperglycemia. Long-term hyperglycemia may result in oxidative stress, damage pancreatic β-cell function and induce insulin resistance. Herein we explored the anti-hypoglycemic effects and mechanisms of action of N-*p*-coumaroyloctopamine (N-p-CO) *in vitro* and *in vivo*. N-p-CO exhibited high antioxidant activity, as indicated by the increased activity of SOD, GSH and GSH-Px in HL-7702 cells induced by both high glucose (HG) and palmitic acid (PA). N-p-CO treatment significantly augmented glucose uptake and glycogen synthesis in HG/PA-treated HL-7702 cells. Moreover, administration of N-p-CO in diabetic mice induced by both high-fat diet (HFD) and streptozotocin (STZ) not only significantly increased the antioxidant levels of GSH-PX, SOD and GSH, but also dramatically alleviated hyperglycemia and hepatic glucose metabolism in a dose-dependent manner. More importantly, N-p-CO upregulated the expressions of PI3K, AKT and GSK3β proteins in both HG/PA-induced HL-7702 cells and HFD/STZ-induced mice. These findings clearly suggest that N-p-CO exerts anti-hypoglycemic and anti-oxidant effects, most probably via the regulation of a PI3K/AKT/GSK3β signaling pathway. Thus, N-p-CO may have high potentials as a new candidate for the prevention and treatment of diabetes.

## 1 Introduction

Diabetes is a chronic metabolic disorder characterized by persistent dysregulation of glucose and lipid metabolism (Huang et al., 2023; Hu et al., 2024). Currently, type 2 diabetes (T2DM) accounts for approximately 90% of the global diabetes cases, which significantly exacerbates physical and economic burdens for individuals (Kane et al., 2018). More and more evidences have suggested that diabetes is a multifactorial disease accompanied by the dysregulation of lipid and glucose metabolism, and oxidative stress, which plays an important role in the occurrence and development of diabetes (Maria and Mattias, 2017; Hu et al., 2020; Sourbh and Jeena, 2022). The long-term hyperglycemia in patients with diabetes may lead to chronic injury and dysfunction of various tissues (Satman et al., 2023; Min et al., 2024). Although the commonly used hypoglycemic clinical agents, such as metformin and glibenclamide can lower blood glucose, they often induce a range of side effects including diarrhea, intestinal flatulence, hemolytic anemia, lactic acidosis and congestive heart failure (Xing et al., 2021; Elkhalifa et al., 2024). Therefore, it is imperative to develop novel hypoglycemic agents with low adverse effects.

**FIGURE 1:**
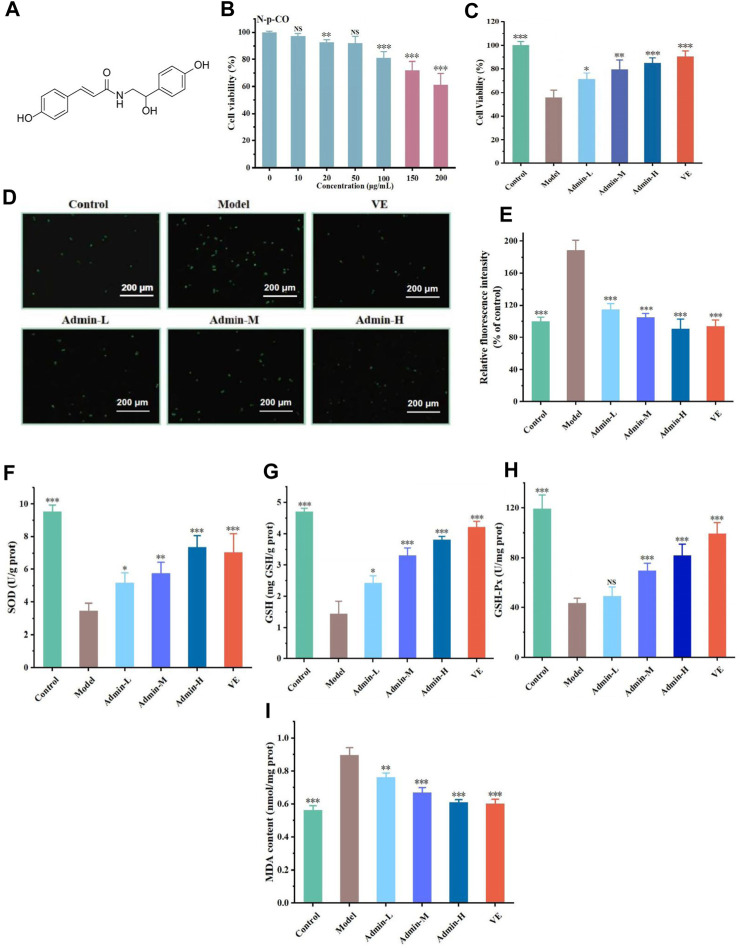
N-p-CO treatment increased the antioxidant capacity in HL-7702 cells exposed to both HG and PA. **(A)** Structure of N-p-CO; **(B)** Cytotoxicity of N-p-CO on HL-7702 cells; **(C)** Effects of different concentrations of N-p-CO on the viability of HG/PA-induced HL-7702 cells; **(D)** Representative images of ROS fluorescence staining; **(E)** Relative fluorescence intensity of ROS; **(F–I)** Contents of SOD, GSH, GSH-Px and MDA in HG/PA-induced HL-7702 cells. Data were represented as mean ± SD (*n* = 3). ^*^ *p* < 0.05, ^**^ *p* < 0.01 and ^***^ *p* < 0.001 compared with the model group.

It is known that oxidative stress has been regarded as a pathological mechanism that contributes to the initiation and progression of T2DM and related complications (Jiang et al., 2020; Argaev-Frenkel and Rosenzweig, 2023). It is a cytotoxic consequence of excessive reactive oxygen species (ROS) production and inhibition of ROS elimination in antioxidant defense systems (Hasandeep et al., 2023). Chronic long-term exposure of hyperglycemia may result in oxidative stress, activate several pathways including protein kinase C (PKC), phosphatidylinositol 3-kinase (PI3K) and serine/threonine protein kinase AKT, and facilitate the formation of advanced glycation end-products (AGEs). The activation of these signaling pathways eventually alters gene expressions and physiological metabolism, leading to organ dysfunction (Hasandeep et al., 2023). Liver is the major regulator for carbohydrate metabolism and lipid biosynthesis (Adeva-Andany et al., 2016). In the setting of T2DM, the disordered oxidation of these substrates can induce excessive levels of systemic glucose and lipid and oxidative stress. These alterations are also accompanied by the disturbance in the metabolism of hepatocellular glucose. Thus, targeting hepatic glucose and glycogen metabolism has been considered as a strategy for the treatment of T2DM (Rines et al., 2016). Several studies have reported that remission of oxidative stress by natural products from plants, such as polyphenols, polysaccharides and flavones, contributes to the improvement of glycolipid metabolism and diabetic phenotype (Chen et al., 2022; He et al., 2022; Zhou et al., 2023). As a naturally-occurring hydroxycinnamoyl amide compound, N-p-coumaroyloctopamine (N-p-CO, Figure 1A) widely exists in alliums, eggplants, potatoes and the like (Hisashi et al., 1998). N-p-CO-enriched allium crude extracts have been found to effectively inhibit the activity of α-glucosidase *in vitro* (Jeppe et al., 2014). In addition, N-p-CO exhibits excellent antioxidant activity (Ayanlowo et al., 2020) and anti-inflammatory activity to reduce the levels of inflammatory cytokines in lipopolysaccharide (LPS)-induced human peripheral blood mononuclear cell (Nchiozem-Ngnitedem et al., 2020). Both biological effects are helpful for the mitigation of diabetes. Thus, it is of great significance to reveal the potential anti-diabetic effects and mechanisms of action of N-p-CO.

In the work reported herein, we sought to evaluate the antioxidant and hypoglycemic effects of N-p-CO in both high glucose (HG)/palmitic acid (PA)-induced HL-7702 cells and high-fat diet (HFD)/treptozotocin (STZ)-induced mice, and recovered the PI3K/AKT/GSK3β pathway involved in the alleviation of N-p-CO on hepatic glucose metabolism.

## 2 Materials and methods

### 2.1 Cell culture

HL-7702 cells were originally obtained from the American Type Culture Collection (ATCC), and cultured in Dulbecco’s Modification of Eagle’s Medium (DMEM, Servicebio, Wuhan, China) supplemented with 10% fetal bovine serum (FBS, PAN, German) and 100 U/mL penicillin and 100 μg/mL streptomycin (HyClone, Logan, United States) at 37°C with 5% carbon dioxide in a humidified incubator prior to treatment.

### 2.2 Cell treatment and bioactivity assay

HL-7702 cells were plated in a 96-well plate with a density of 1 × 10^5^ cells per well for 24 h and incubated for an additional 24 h with serum-free medium in combination with or without 30 mM glucose, 0.2 mM PA, or 0–200 μg/mL N-p-CO (Macklin, Shanghai, China). The cytotoxicity of N-p-CO was measured using a 3-[4,5-dimethylthiazol-2-yl]-2,5-diphenyltetrazolium bromide (MTT, Biofroxx, Einhausen, German) assay according to previous methods (Wang et al., 2020). Briefly, after treatment with N-p-CO, MTT (5 mg/mL, 20 μL) was added to each well for 4 h and then dimethyl sulfoxide (DMSO, 150 μL) was added. The absorbance was determined at 490 nm on a Microplate Reader (BioTek, Vermont, United States).

### 2.3 Assessment of cellular glucose consumption and uptake

For the consumption of cellular glucose, after treatment with or without N-p-CO (10 μg/mL, 20 μg/mL and 50 μg/mL) or Metformin (Met, HPLC >97%, Macklin, Shanghai, China; 50 μg/mL), the culture supernatant of HL-7702 cells induced by both PA (0.2 mM) and glucose (30 mM) was collected and quantified using a sulfate-anthrone method. The concentration of glucose was determined using a standard curve of glucose under an absorbance of 620 nm.

The glucose uptake was measured according to a previous method (Ye et al., 2022). In brief, after treatments with N-p-CO of varying concentrations, the HL-7702 cells were washed with PBS (Adamas life, Shanghai, China) and then incubated with fluorescent deoxyglucose derivative (2-NBDG, Invivochem, Guangzhou, China; 100 μM) at 37°C for 30 min. Subsequently, fluorescence microscopy (Olympus, Nagano, Japan) was used to detect cellular fluorescence intensity.

### 2.4 Determination of cell glycogen

The content of glycogen in cells was determined using the method described before (Huang et al., 2015). Briefly, cells supernatant was air-dried naturally and the mass was quantified. A solution containing 30% KOH (Aladdin, Shanghai, China) was added and heated in a water bath for 30 min. Then anhydrous ethanol (1.5 mL) was added and centrifuged at 12,000 g for 15 min at 4°C. The glucose content was determined using a sulfuric acid-anthrone (Macklin, Shanghai, China) method.

### 2.5 Animal model and experimental design

6–8 Week-old C57BL/6 healthy male mice (20 ± 2 g) were obtained from BesTest Biotechnology Co., LTD (Zhuhai, Guangdong, China) and housed under standard specific pathogen-free (SPF) conditions with a constant temperature of 25°C and a 12-h light/dark cycle. To induce T2DM, the mice were adapted for 7 days and treated with both high-fat and high-sugar diets (D12492, Xiao Shu You Tai, Beijing, China) and intraperitoneal STZ (Biofiven, Guangzhou, China). The mice in a control group received a normal diet. The mice in the other groups were fed with a high-fat and high-sugar diet. The mice in the experimental groups were fasted for 12 h and intraperitoneally injected with 40 mg/kg STZ dissolved in sodium citrate buffer solution (pH 4.4) (Xu et al., 2020). The mice in normal control group was administered with an equal volume of citric acid-sodium citrate buffer solution instead of STZ injection. Mice with T2DM were identified using fasting blood glucose levels at ≥ 16 mmol/L after 5 days and selected for further treatment. Diabetic mice were randomly divided into four groups (*n* = 5), that is, diabetic model group (DM), positive control group (*i.p.* 200 mg/kg metformin, DM-Met), low-dose group (*i.p.* 5 mg/kg N-p-CO, DM-L) and high-dose group (*i.p.* 10 mg/kg N-p-CO, DM-H). The mice in normal control and diabetic model groups received an equal volume of normal saline for 5 weeks.

### 2.6 Oral glucose tolerance test (OGTT)

After treatment for 5 weeks, mice were fasted overnight and given 2 g/kg glucose by oral gavage. The blood glucose of mice was measured with a blood glucose meter (Sinocare, Shenzhen, China) after glucose administration for 0, 30, 60, 90, and 120 min. The area under the curve (AUC) was calculated to give the OGTT of mice.

### 2.7 Oxidation detection

The antioxidant activity of N-p-CO was assessed by measuring the contents of ROS, superoxide dismutase (SOD), glutathione (GSH), glutathione peroxidases GSH-px and malondialdehyde (MDA). For the detection of cellular antioxidant activity, the level of ROS was measured by using a 2,7-dichlorodihdrofluorescein diacetate (DCFH-DA) probe staining method in lined with previous reports (Sun et al., 2021; Zhang et al., 2021). Briefly, HL-7702 cells were plated in a 96-well plate with a density of 1 × 10^5^ cells per well for 24 h and incubated for an additional 24 h with serum-free medium, 30 mM glucose, 0.2 mM PA, or 0, 10, 20 and 50 μg/mL N-p-CO (Ye et al., 2024). The content of ROS was measured using a ROS detection kit and observed with fluorescence microscope.

For the detection of SOD, GSH, GSH-px and MDA in cells and liver, cell culture supernatant or heptic homogenate was determined by using commercial reagent kits (Nanjing Jiancheng Bioengineering Institute, Nanjing, China) following the manufacturer’s instructions with mild modifications (Sun et al., 2020; Zhang et al., 2021).

### 2.8 Serum biochemical analysis

Serum and hepatic levels of alanine aminotransferase (ALT), aspartate transaminase (AST) and glycated serum protein (GSP) were detected by commercial assay kits in line with the manufacturer’s protocol with mild adjustments (Qi et al., 2022).

### 2.9 Histopathological examination

Histopathological examination of liver was performed with a standard process with mild modifications (Ma et al., 2023; Zhang et al., 2023). The liver tissue from the mice was fixed with 4% paraformaldehyde solution for 24 h, dehydrated with gradient ethanol solution and embedded in paraffin. Sections (4 µm) were prepared and stained with hematoxylin and eosin (H&E) and periodic acid-schiff (PAS) staining, respectively. The morphology was obtained with a light microscope (Olympus, Nagano, Japan).

### 2.10 Western blot assay

The western blot assay was performed using the procedures previously described (Tao et al., 2020; Wang et al., 2021; Sun et al., 2023). The total protein of liver or cells was extracted with the RIPA lysis buffer and quantified with a BCA protein detection kit (Aidlab, Beijing, China). Each sample was loaded with 1 μL protein, separated with 10% SDS-PAGE and transferred onto a PVDF membrane (Millipore, Massachusetts, United States). Membrane binding proteins were blocked with 10% milk (Yili, Neimenggu, China) at room temperature for 1 h. Subsequently, primary antibodies including AMPK (1:1,000, Cell Signaling Technology, MA, United States), GLUT2 (1:1,000, Cell Signaling Technology, MA, United States), PI3K (1:1,000, Cell Signaling Technology, MA, United States), AKT (1:1,000, Cell Signaling Technology, MA, United States), GSK3β (1:1,000, Cell Signaling Technology, MA, United States), p-GSK3β (1:1,000, Cell Signaling Technology, MA, United States) and β-actin (1:1,000, Cell Signaling Technology, MA, United States), were incubated overnight at 4°C followed by three washes with Tris-buffered saline Tween-20 (TBST) (Sinopharm, Beijing, China). The membrane was then incubated with a horseradish peroxidase-labeled secondary antibody (1:2000, Sarvicebio, Wuhan, China) at room temperature for another 1 h and washed three times with TBST. Finally, the membrane-bound proteins were detected using an ECL detection reagent (Beyotime, Shanghai, China) and visualized on an Image Quant LAS-4000 digital imaging system (GE Healthcare Bio-Sciences, Uppsala, Sweden).

### 2.11 qPCR analysis

The relative expressions of genes were conducted according to established protocols (Wang and Mu, 2021; Hao et al., 2022; Liu et al., 2022). Total RNA from the cells was extracted using a Trizol extraction reagent (Biosharp, Guangzhou, China). The RNA was reversed into c-DNA using an RT reagent kit (ThermoFisher, Massachusetts, United States). qRT-PCR was performed using a TB Green kit (Servicebio, Wuhan, China) on the Applied Biosystems Step One RT-PCR system (Applied Biosystems, Forster city, United States). The primer sequences of the target genes are listed in Table 1.

**TABLE 1:** Primers for the PCR assay.

Name	Forward primer (5′-3′)	Reverse primer (5′-3′)
GSK3β	GAC​AGT​GGT​GTG​GAT​CAG​TTG​GTG	GCG​ATT​GCC​TCT​GGT​GGA​GTT​C
GLUT2	CAC​CAG​CAC​ATA​CGA​CAC​CAG​AC	CCC​AAG​CCA​CCC​ACC​AAA​GAA​C
PI3K	AGG​ATG​CCC​AAC​TTG​ATG​CTG​ATG	CCC​GTT​CAT​ATA​GGG​TGT​CGC​TGT​G
AMPKα	CAA​CTA​TCG​ATC​TTG​CCA​AAG​G	AAC​AGG​AGA​AGA​GTC​AAG​TGA​G
AKT	TGA​CCA​TGA​ACG​AGT​TTG​AGT​A	GAG​GAT​CTT​CAT​GGC​GTA​GTA​G
β-Actin	ATG​TGG​ATC​AGC​AAG​CAG​GA	ATG​TGG​ATC​AGC​AAG​CAG​GA

### 2.12 Statistical analysis

Data are presented as mean ± standard deviation. One-way analysis of variance (ANOVA) was performed to analyze the significant differences among groups by IBM SPSS Statistics 26.0 (SPSS Inc., Chicago, United States). Results were visualized using Origin 2022 software (OriginLab, Electronic Arts Inc., United States). Statistical significance is indicated by ^*^ *p* < 0.05, ^**^ *p* < 0.01 and ^***^ *p* < 0.001.

## 3 Result

### 3.1 N-p-CO ameliorated oxidative stress of HG/PA-induced HL-7702 cells

To assess the antioxidant capacity of N-p-CO, a model of HL-7702 cells was established in the absence or presence of HG/PA. The concentrations of 10 μg/mL, 20 μg/mL and 50 μg/mL without any effect on the cell viability were selected for subsequent experiments according to the cytotoxicity induced by N-p-CO in HL-7702 cells (Figure 1B). Exposure of HL-7702 cells to N-p-CO led to a significant decrease in the cell viability in a dose-dependent manner. The viability was significantly decreased to 81.02% ± 4.65% at the concentration of 50 μg/mL of HG/PA (Figure 1B). Compared with the cell viability of HL-7702 cells exposed to HG/PA, treatment with N-p-CO significantly increased the viability of HG/PA-induced cells in a dose-dependent manner (Figures 1C–I). Furthermore, ROS fluorescence staining showed that the content of intracellular ROS in the model group was significantly higher than that in the control group. Supplementation with N-p-CO significantly reduced the ROS levels elevated by HG/PA in a dose dependent manner (Figures 1D, E). N-p-CO also significantly increased the levels of SOD, GSH and GSH-px, and decreased the contents of MDA in HG/PA-incued HL-7702 cells in a dose dependent manner (Figures 1F–I). These results indicate that N-p-CO has the potential to alleviate HG/PA-induced oxidative damage.

### 3.2 N-p-CO increased glucose uptake of HL-7702 cells induced by HG/PA

**FIGURE 2:**
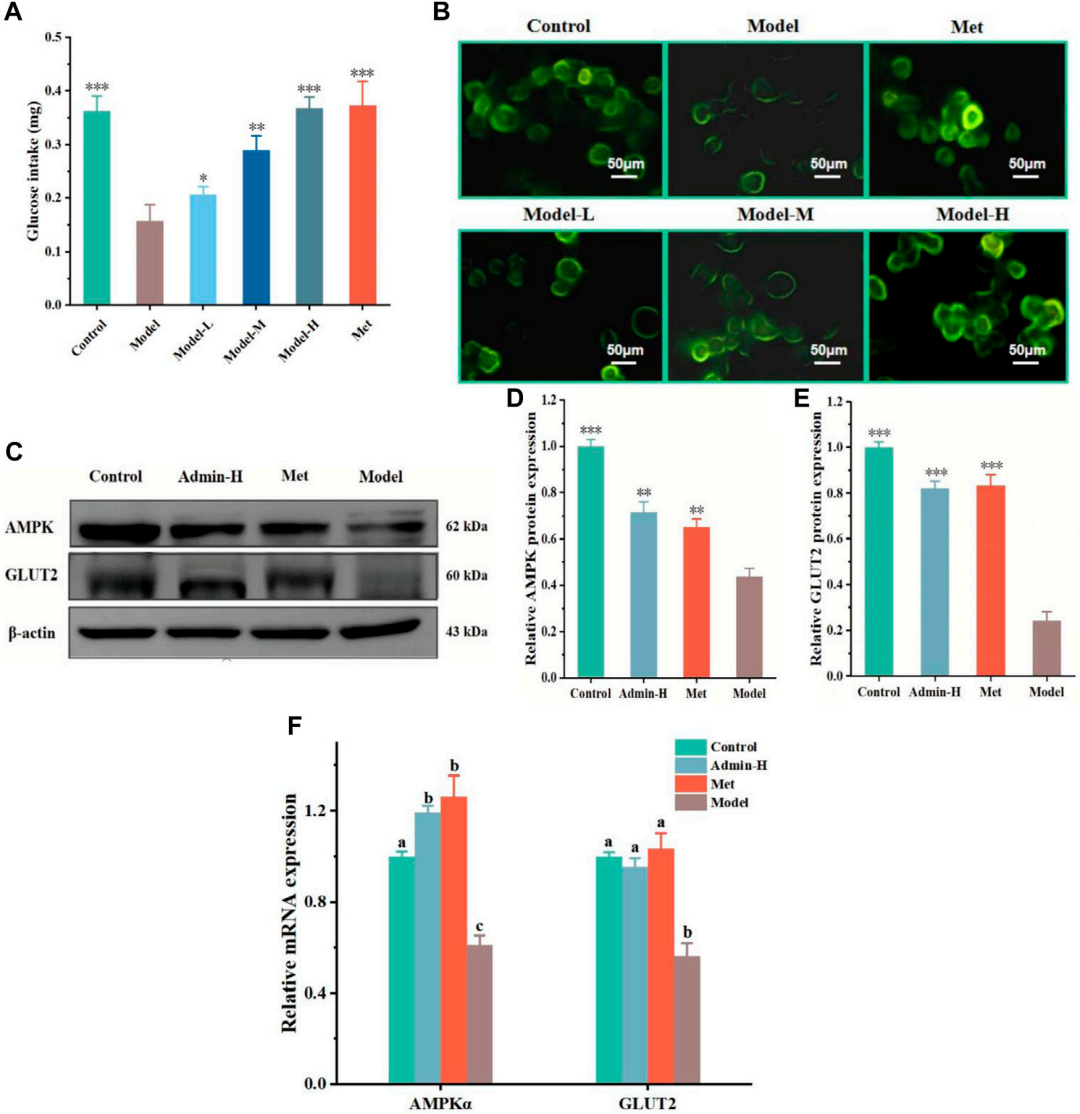
N-p-CO increases cellular glucose uptake in HG/PA-induced HL-7702 cells. **(A)** The glucose uptake of HG/PA-induced HL-7702 cells was determined by an anthrone-sulfuric method; **(B)** Representative images of 2-NBDG fluorescence staining; **(C)** Representative bands of GLUT2 and AMPK determined by western blot; **(D,E)** Relative expressions of AMPK and GLUT2 proteins; **(F)** Relative mRNA expression of AMPKα and GLUT2. Data were represented as mean ± SD (*n* = 3). ^*^ *p* < 0.05, ^**^ *p* < 0.01 and ^***^ *p* < 0.001 compared with the model group.

High sugar exposure can promote the generation of ROS and in turn activates AMPKα to reduce the cellular glucose uptake capacity, eventually resulting in metabolic disorders (Jiang et al., 2021). Glucose uptake was measured, with met as the positive control drug which had been a clear mechanism that met can increase GLUT2 expression. The glucose uptake capacity of HG/PA-treated HL-7702 cells was significantly lower than that of the control group. The glucose uptake capacity of HL-7702 cells was significantly improved after treatment with metformin and different doses of N-p-CO (Figures 2A, B). As cellular glucose uptake is mainly regulated by GLUT2 (Rathinam and Pari, 2016), we conducted western blot analysis to observe the effects of N-p-CO on the expression of GLUT2 protein. Compared with the control cells, cells exposed to HG/PA presented a clear downregulation of GLUT2 protein. Treatment with N-p-CO or an AMPK agonist metformin (Sun Y. et al., 2023) significantly upregulated the expression of GLUT2 in the HG/PA-treated HL-7702 cells. Interestingly, the upregulation of GLUT2 and the enhancement of glucose uptake capacity by N-p-CO were significantly reversed by metformin treatment. This result suggests that N-p-CO effectively increases glucose uptake, which may be related to the upregulation of GLUT2 and the activation of AMPK (Figures 2C–F).

### 3.3 N-p-CO upregulated the PI3K/AKT/GSK3β pathway and increased the content of glycogen in HG/PA-induced HL-7702 cells

**FIGURE 3:**
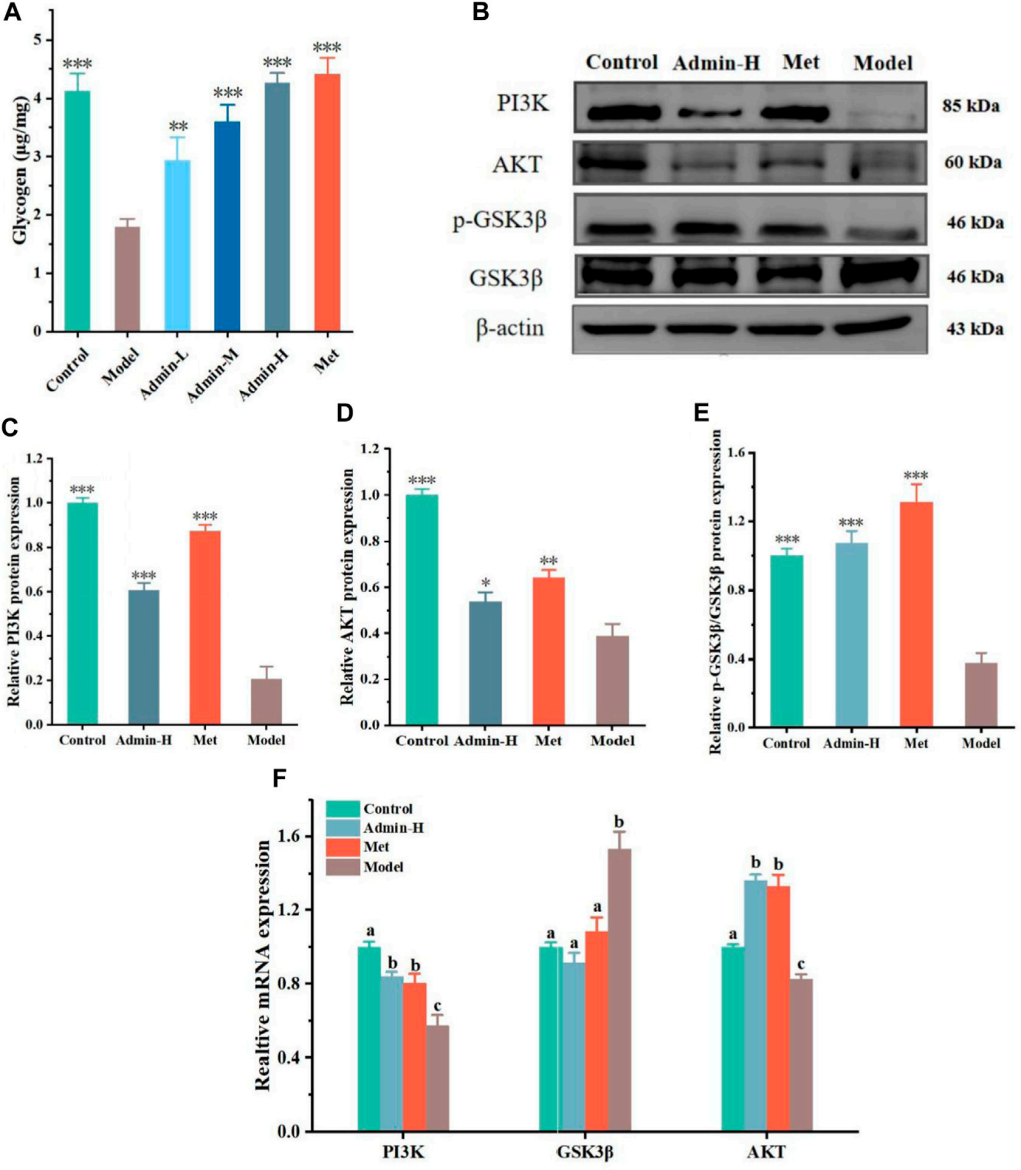
N-p-CO promoted glycogen production related to the PI3K/AKT/GSK3β pathway in HG/PA-induced HL-7702 cells. **(A)** Content of glycogen in HG/PA-induced HL-7702 cells; **(B)** Representative western blot bands of PI3K, AKT and GSK3β proteins; **(C–E)** The relative expressions of PI3K, AKT and GSK3β proteins; **(F)** Relative mRNA expression of PI3K, AKT and GSK3β. Data were represented as mean ± SD (*n* = 3). ^*^ *p* < 0.05, ^**^ *p* < 0.01 and ^***^ *p* < 0.001 compared with the model group.

As the uptake of glucose is mainly stored in the form of glycogen in liver (M. et al., 2016), we next tested whether N-p-CO could affect the generation of glycogen in hepatocytes. It was observed that the content of glycogen in HG/PA-induced HL-7701 cells was significantly lower than that of controls. Treatment with N-p-CO could effectively increase the content of intracellular glycogen in HG/PA-induced HL-7701 cells when compared with the model group (Figure 3A). The production of glycogen has been suggested to be associated with the inhibition of glycogen synthase kinase 3β (GSK3β) (Yan et al., 2023). Then we characterized the protein expressions of GSK3β involved signaling pathway using western blot. Significant downregulations of p-GSK3β proteins and its upstream gene PI3K and AKT were observed in the model group when compared with the control group. After intervention with N-p-CO, the expressions of these proteins were significantly upregulated in HG/PA-induced HL-7701 cells (Figures 3B–F). These findings reveal that N-p-CO could activate the PI3K/AKT/GSK3β pathway and increase the content of glycogen in HG/PA-induced HL-7702 cells, which may hold great potentials for the mitigation of hyperglycemia.

### 3.4 N-p-CO decreased blood glucose and improved physical phenotypes in HFD/STZ-induced T2DM mice

**FIGURE 4:**
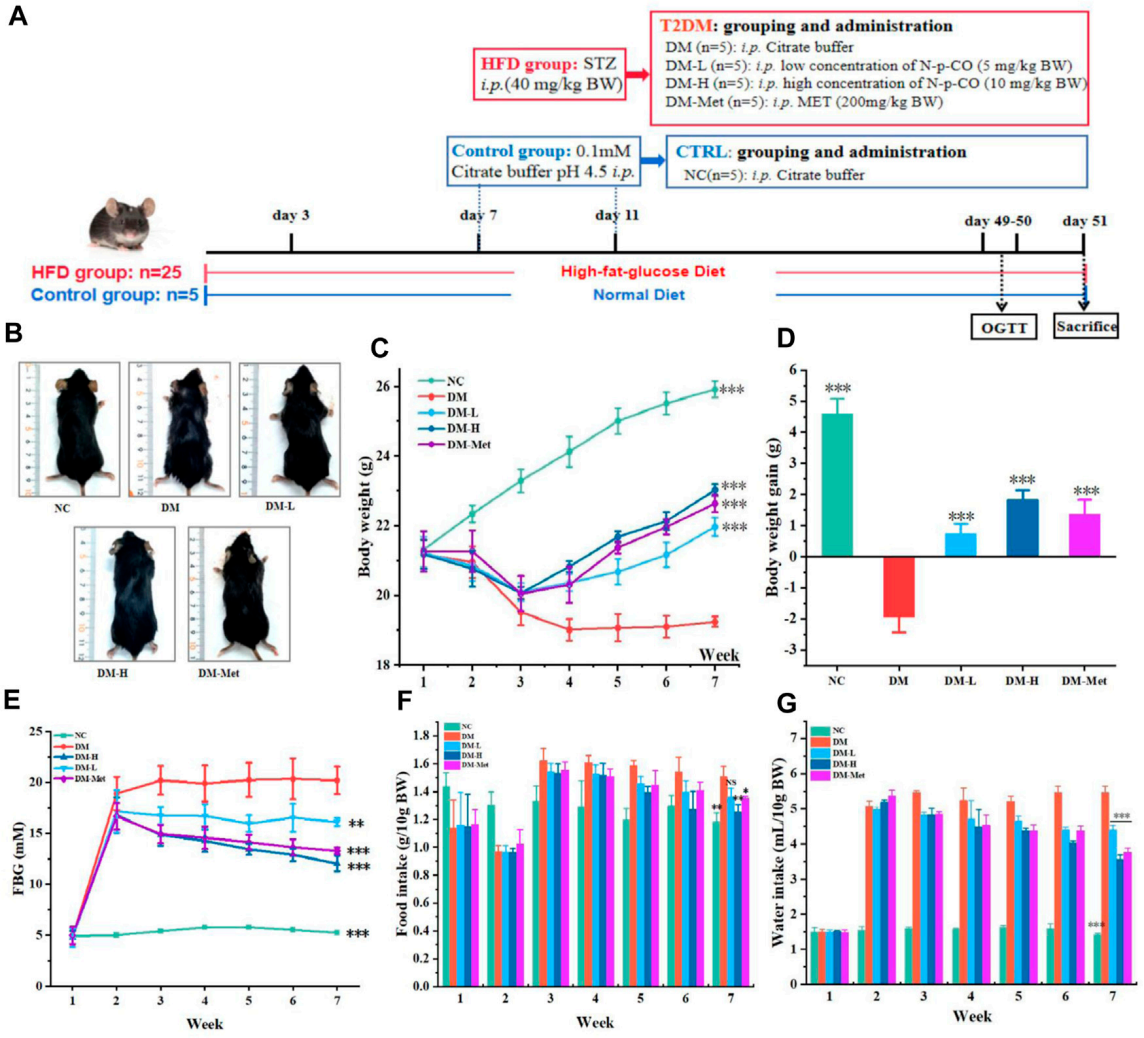
N-p-CO improved the phenotypes of hyperglycemia in HFD/STZ-induced mice. **(A)** Experimental process and employed groups; **(B)** Representative images of mice; **(C)** The changes in the body weight of mice; **(D)** The body weight gain of mice; **(E)** The changes of fasting blood glucose of mice; **(F)** Food intake of mice; **(G)** Water intake of mice. Data were represented as mean ± SD (*n* = 5). ^*^ *p* < 0.05, ^**^ *p* < 0.01 and ^***^ *p* < 0.001 compared with the DM group.

Because administration of N-p-CO could ameliorate glucose uptake and glycogen production *in vitro*, we then tested whether N-p-CO exhibited these effects in mice treated with HFD/STZ. Mice were treated with HFD/STZ with or without N-p-CO for 8 weeks (Figure 4A). It could be found that mice in the DM group exhibited significantly reduced sizes, disheveled and dull hair (Figure 4B). As expected, these physical appearances of DM mice characterized by soft and lustrous hair, were significantly restored after intervention with N-p-CO or Met (Figure 4B). Compared with the control mice, HFD/STZ treatment induced an obvious decrease in the body weight of mice in the DM group, while relatively slow decline in mice administrated with either N-p-CO or Met (Figures 4C, D). Furthermore, the fasting blood sugar (FBG) of the HFD/STZ-induced mice was significantly higher than that in the NC group. Compared with the DM group, treatment with N-p-CO significantly reduced the level of FBG in HFD/STZ-induced mice in a dose-dependent manner (Figure 4E). Additionally, DM mice displayed significantly increased consumption of water and food when compared with the controls, which was improved after treatment with either N-p-CO or Met (Figures 4F, G). These results indicate that N-p-CO could effectively improve the phenotypes of hyperglycemia in mice with diabetes induced by HFD/STZ.

### 3.5 N-p-CO enhanced antioxidant ability in mice treated by HFD/STZ

**FIGURE 5:**
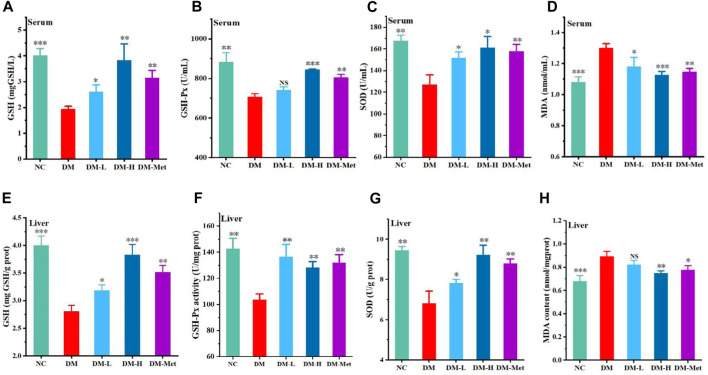
N-p-CO enhanced systemic and hepatic antioxidant ability in HFD/STZ-induced mice. **(A–C)** The levels of GSH, GSH-Px and SOD in serum; **(D)** The content of MDA in serum; **(E–G)** The hepatic levels of GSH, GSH-Px and SOD; **(H)** The content of MDA in liver. Data were represented as mean ± SD (*n* = 3). ^*^ *p* < 0.05, ^**^ *p* < 0.01 and ^***^ *p* < 0.001 compared with the DM group.

As the above data have shown that N-p-CO supplementation could prevent oxidative damage induced by HG/PA in liver cells, the antioxidant effect of N-p-CO was further determined in mice treated by HFD/STZ. Compared with mice in the NC group, mice treated with HFD/STZ presented a significant decrease in the levels of GSH, GSH-Px and SOD in the serum. Administration with N-p-CO significantly increased the serum levels of GSH, GSH-Px and SOD in mice induced by HFD/STZ (Figures 5A–C). The content of MDA in serum of DM group was significantly higher than that in the control groups. Compared with the DM group, treatment with N-p-CO reduced the serum content of MDA in HFD/STZ-induced mice. Meanwhile, the hepatic levels of GSH, GSH-Px and SOD were significantly decreased in the mice induced by HFD/STZ when compared with the NC group. The levels of these antioxidant enzymes in the liver of HFD/STZ-induced mice were remarkably increased by N-p-CO treatment in a dose-dependent manner (Figures 5E–G). Compared with the NC group, HFD/STZ-induced mice displayed evaluated content of MDA in the liver, which was significantly decreased after treatment with N-p-CO (Figure 5H). These results suggest that N-p-CO could effectively enhance the antioxidant capacity of mice induced by HFD/STZ.

### 3.6 N-p-CO improved liver function in HFD/STZ-induced mice

**FIGURE 6:**
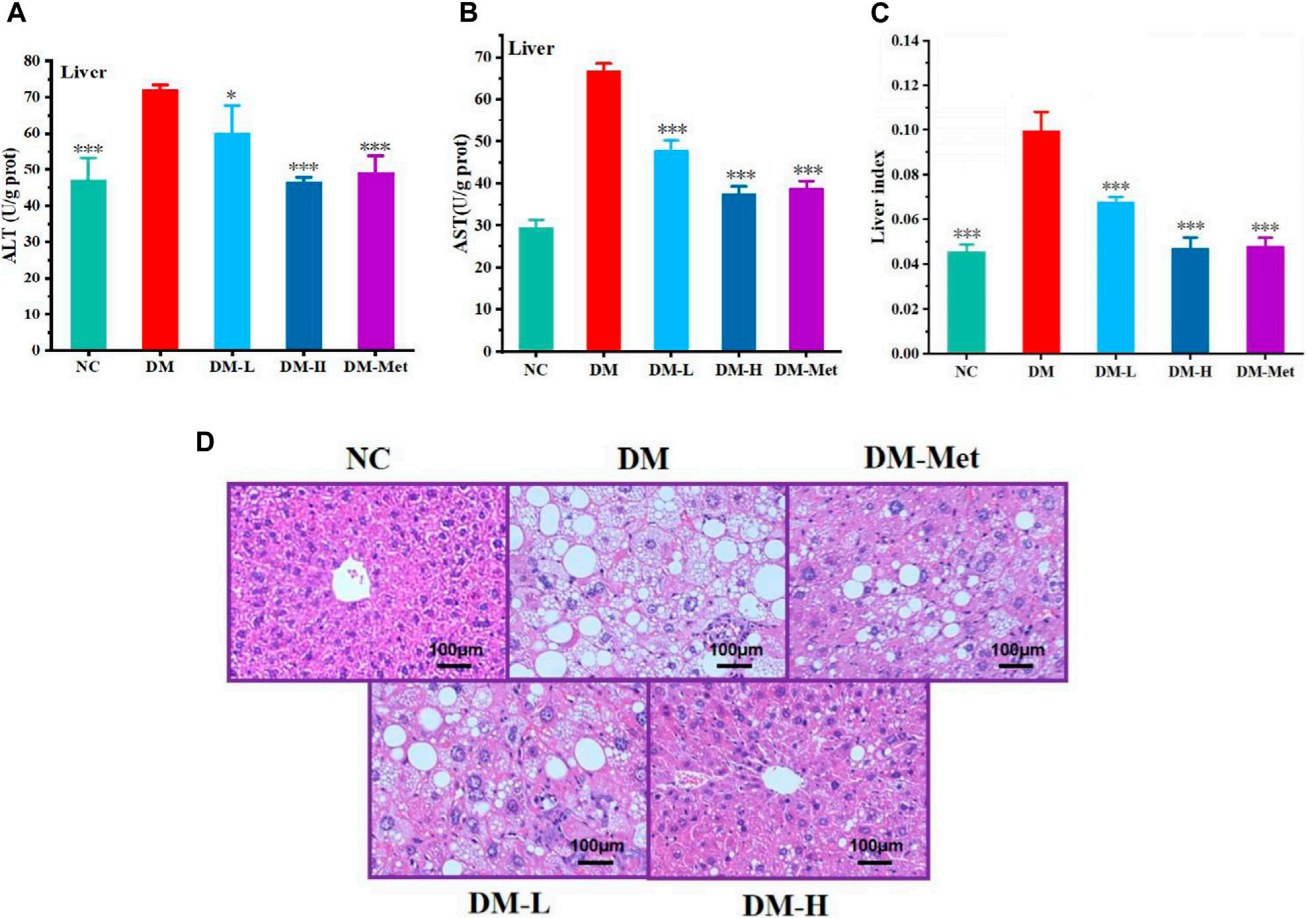
N-p-CO improved the function of liver in HFD/STZ induced mice. **(A)** Content of ALT; **(B)** Content of AST; **(C)** Liver index; **(D)** H&E staining of liver. Bars represent mean ± SD (*n* = 5). ^*^ *p* < 0.05, ^**^ *p* < 0.01 and ^***^ *p* < 0.001 compared with the DM group.

As liver acts as a vital organ involved in the metabolism of glucose, we evaluated the protective effect of N-p-CO on liver function and morphology. Compared with the NC group, the ratio of liver weight and body weight, and the levels of ALT and AST in the liver of mice induced by HFD/STZ were significantly increased. N-p-CO treatment significantly reduced liver index and the hepatic levels of ALT and AST in HFD/STZ-induced mice (Figures 6A–C). The H&E staining of liver showed that hepatocytes exhibited intact architecture with well-defined boundaries, uniform sizes, and abundant cytoplasm in liver from the NC group. The hepatic sinuses displayed a regular arrangement with clear sinusoidal spaces occasionally accompanied by a few inflammatory cells in the control mice. Conversely, in relative to the control mice, the hepatocytes in the DM group showed hypertrophy, vacuolar degeneration, and cytoplasmic accumulation of numerous round lipid droplets of varying sizes (Figure 6D). Moreover, there was significant infiltration of inflammatory cells, hepatocyte necrosis along with indistinct hepatic lobular boundaries and disorganized arrangement of hepatic cords. Additionally, a great number of red blood cells were observed within the hepatic cords, indicating severe cellular damage. N-p-CO treatment induced a significant reduction in hepatic steatosis and improvement of glomerular morphology in the HFD/STZ-induced mice. These findings indicate that N-p-CO may have high potentials in improving hepatic function and metabolic activity, which may contribute to the remission of diabetes.

### 3.7 N-p-CO activated PI3K/AKT/GSK3β pathway and improved the metabolism of hepatic glucose in mice

**FIGURE 7:**
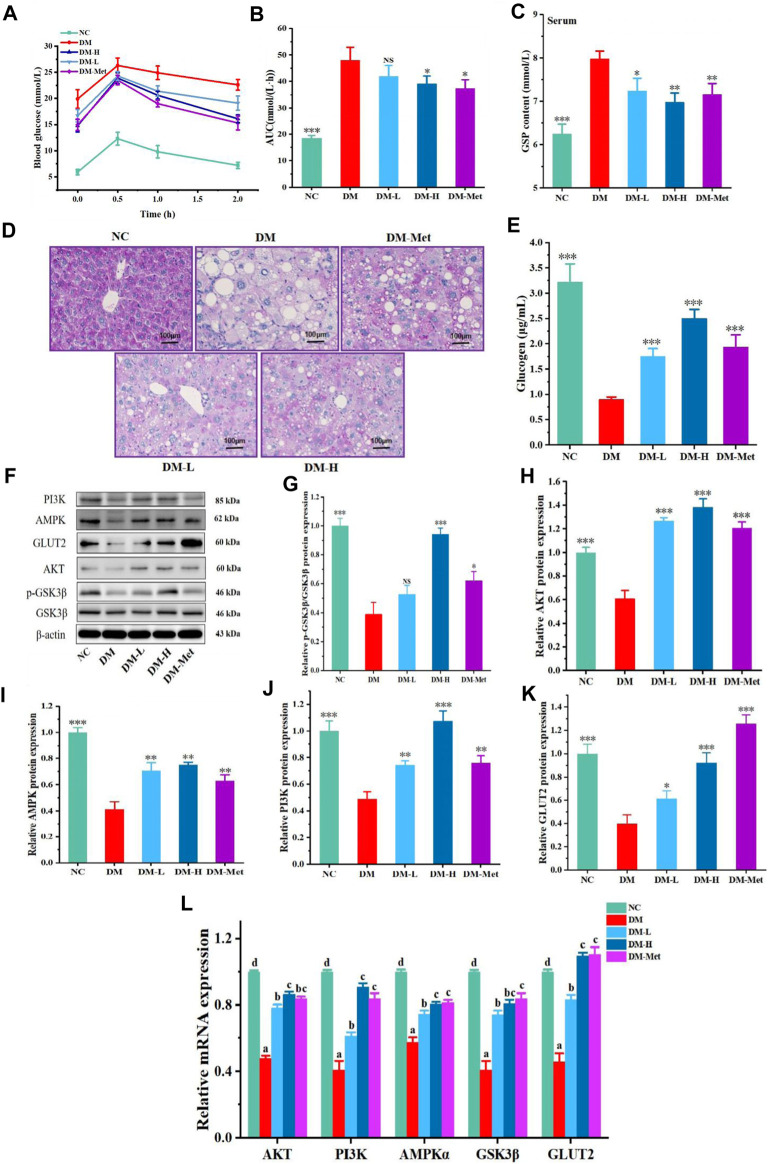
N-p-CO improved hepatic glucose metabolism and activated a PI3K/AKT/GSK3β pathway in HFD/STZ-treated mice. **(A)** The curve of oral glucose tolerance; **(B)** AUC of blood glucose; **(C)** Content of GSP in serum; **(D)** PAS staining of liver; **(E)** Content of glucogen in liver; **(F)** Representative western blot bands of PI3K, AKT, GSK3β, AMPK and GLUT2 proteins; **(G–K)** Relative expressions of PI3K, AKT, GSK3β, AMPK and GLUT2 proteins; **(L)** Relative mRNA expression of AKT, PI3K, AMPKα, GSK3β and GLUT2. Data were represented as mean ± SD (*n* = 5). ^*^ *p* < 0.05, ^**^ *p* < 0.01 and ^***^ *p* < 0.001 compared with the DM group.

The above results indicated that N-p-CO could improve the function of liver in mice induced by HFD/STZ and glucose metabolism in HL-7702 cells induced by HG/PA. We next evaluated whether N-p-CO could also improve the metabolism of glucose in the liver of diabetic mice. A significant increase in the level of blood glucose was found in HFD/STZ-induced mice when compared with the mice in NC group, suggesting that HFD/STZ treatment induced an impaired glucose tolerance in mice. Oral glucose tolerance was significantly improved by the decrease in the blood glucose level by administration with either Met or N-p-CO in HFD/STZ-induced mice (Figures 7A, B). Serum level of GSP in HFD/STZ-induced DM mice was significantly higher than that in the NC group. Supplementation with either N-p-CO or Met significantly reduced the content of GSP in mice induced by HFD/STZ (Figure 7C). Furthermore, glycogen production in liver was determined using periodic PAS staining. The content of glycogen in liver in HFD/STZ-induced DM mice was decreased when compared with the NC group. Administration with either N-p-CO or Met significantly increased the production of glycogen in the liver of mice induced by HFD/STZ (Figures 7D, E). Importantly, in line with the changes in cells, the downregulations of hepatic PI3K, AKT and GSK3β expressions by HFD/STZ exposure were significantly restored in mice by treatment with either N-p-CO or Met. Notably, HFD/STZ exposure caused a clear downregulation of hepatic GLUT2 protein in mice, which was significantly increased after treatment with either N-p-CO or Met. These findings suggest that administration with N-p-CO effectively improved hepatic glucose metabolism, which may be related to the activation of a PI3K/AKT/GSK3β pathway in mice (Figures 7F–L).

## 4 Discussion

It has been suggested that chronic or intermittent hyperglycemia is linked to the development of diabetic complications (Volpe et al., 2018; Wu et al., 2023). Several signaling pathways involved in diabetic complications may be directly triggered by hyperglycemia in different tissues, due to the production of ROS, the formation of AGEs and the secretion of pro-inflammatory cytokines (Song et al., 2022; Yoshikawa et al., 2022). Thus, reduction of ROS has been proposed as an effective strategy for mitigating inflammation and complications associated with diabetes (Cheng et al., 2019). Several natural antioxidants including polyphenols, have been shown to reduce the risk of T2DM via increasing the antioxidant defense, regulating inflammatory response and decreasing blood glucose and insulin resistance (Shah et al., 2022; Kukavica et al., 2024). Therefore, it is in need to further discover high-efficiency, low-toxicity and multi-target antioxidants for diabetes and its complications. In this study, we found that as a natural polyphenol compound, N-p-CO exhibited strong antioxidant ability and hypoglycemic effect in both HG/PA-induced HL-7702 cells and HFD/STZ-induced mice. Furthermore, supplementation with N-p-CO significantly activated the hepatic PI3K/AKT/GSK3β signaling pathway, which may participate in the metabolism of glucose in liver. N-p-CO may serve as an antioxidant and regulator of hepatic glucose metabolism in diabetes, which may provide a theoretical basis for the clinical use of N-p-CO.

Liver is an important organ for the maintenance of glucose homeostasis via controlling glycogenesis, glycogenolysis, glycolysis, gluconeogenesis and other pathways (Ding et al., 2018). However, these hepatic processes are dysregulated in diabetes mellitus, which may contribute to high hepatic glucose production and hyperglycaemia (Petersen et al., 2017). It is reported that high glucose can induce apoptosis and oxidative stress injury for different cells, and hepatic glucose and glycogen metabolism have been considered to be related to the hepatic glucose output (Ding et al., 2021). The enhancement of hepatic glucose and glycogen metabolism has been proposed as a potential avenue for the development of anti-diabetic therapy. However, this aspect remains largely unexplored (Rines et al., 2016). The dysregulation of hepatic glucose metabolism probably results from the oxidative damage to enzymes that are related to the glycolysis, tricarboxylic acid cycle and ATP biosynthesis (Jha and Mazumder, 2019). Therefore, targeting oxidative stress to improve the metabolism of glucose in liver has been suggested as a strategy for the treatment of hepatotoxicity, diabetes and its complications (Sun et al., 2020b). For example, dapagliflozin protects hyperglycemia-induced cardiomyocytes damage through inhibiting NADPH oxidase-mediated oxidative stress (Xing et al., 2021). Notably, polyphenols have been considered to have the potential to treat diabetes due to their strong antioxidant ability (Krawczyk et al., 2023). Rutin and quercetin have been found to enhance glucose uptake by decreasing oxidative stress in L6 myotubes induced by tertiary butyl hydrogen peroxide (Dhanya et al., 2014). Resveratrol could afford advantageous effects for glucose uptake and metabolism by activating the AKT pathway and reducing the oxidative injury in the context of insulin resistance related with pre-diabetes and T2DM (Brasnyó et al., 2011).

Consistent with the strong anti-oxidant effects of these polyphenols in T2DM, we found that N-p-CO treatment significantly increased the activity of GSH-PX, SOD and GSH in both HG/PA-induced HL-7702 cells and HFD/STZ-induced mice, indicating a lowered degree of the hydroxyl free radical production induced by N-p-CO. In other words, the strong antioxidant properties of N-p-CO may result from the ability to activate antioxidant enzymes and other mechanisms. Notably, administration with N-p-CO not only dramatically augmented glucose uptake and glycogen synthesis in HG/PA-treated HL-7702 cells, but also significantly alleviated hyperglycemia and hepatic glucose metabolism in a dose-dependent manner in diabetic mice induced by HFD/STZ. This result suggests that the consumption of N-p-CO could effectively improve hepatocellular glucose homeostasis in T2DM. These findings also demonstrate that the amelioration of dysregulation of hepatic glucose metabolism by N-p-CO was, at least in part, owing to the increase in the antioxidant defense.

**FIGURE 8:**
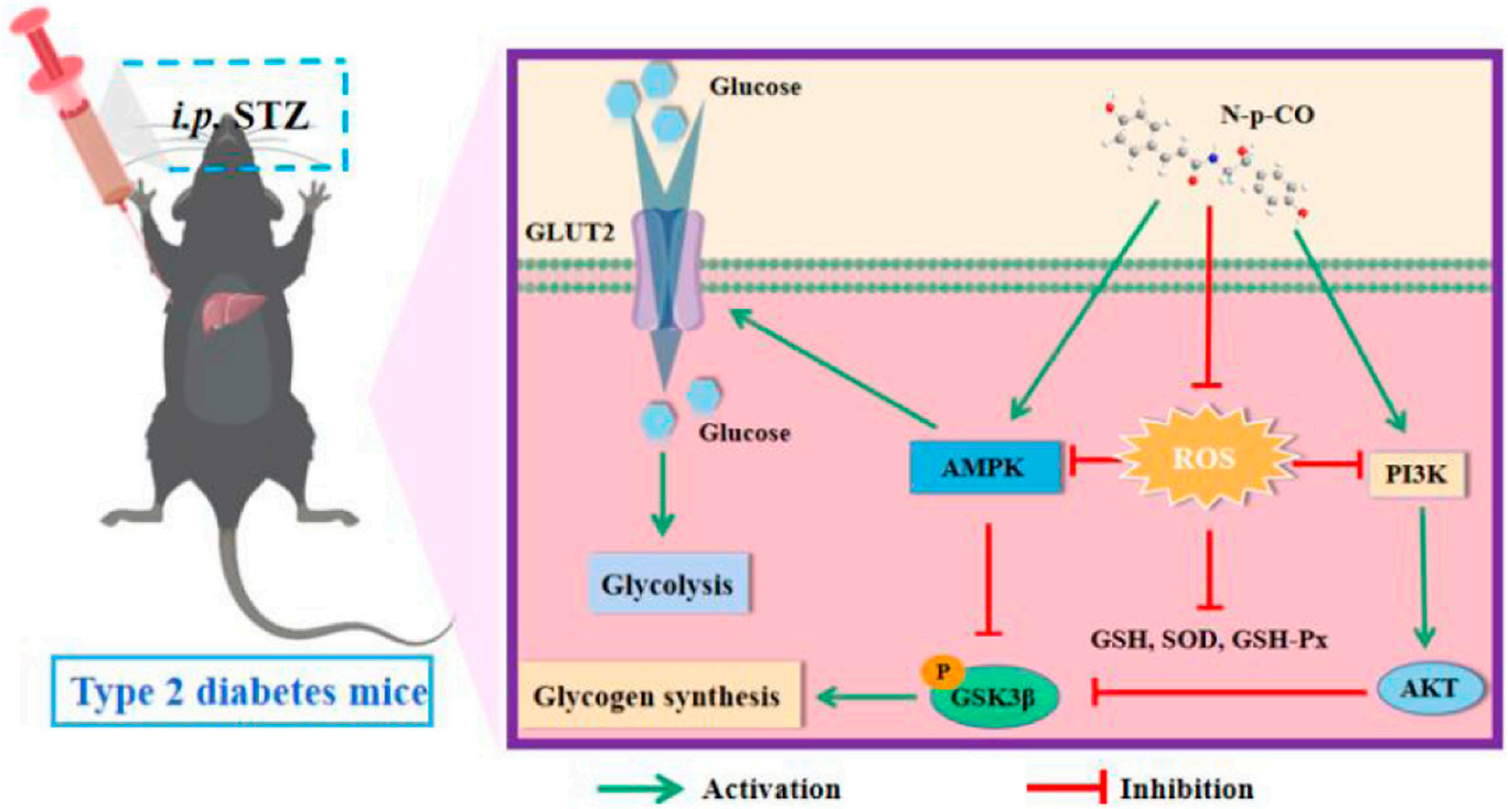
Hypoglycemic effects and possible mechanisms of N-p-CO *in vivo* and *in vitro*.

The regulation of hepatic glucose and lipid metabolism is controlled by the intrinsic molecular signaling pathways, such as PI3K/AKT, MAPK and AMPK pathways (Kumar et al., 2021). For instance, the PI3K pathway has been identified as a critical pathway for the actions of insulin in liver, which can be differentially regulated by AKT and PKC λ/ξ and induced differential actions of insulin and PI3K (Taniguchi et al., 2006). The PI3K-dependent activation of AKT is helpful for the regulation of hepatic glucose metabolism induced by insulin. Impaired phosphorylation of AKT and its substrate GSK3β suppresses gluconeogenesis and increases glycogen synthesis (Ge et al., 2015). Previous studies have reported that upregulation of PI3K/AKT and AMPK proteins by polyphenols is able to inhibit intestinal glucose absorption by sodium-dependent glucose transporter 1 (SGLT1), enhance insulin-dependent glucose uptake, activate PI3K-dependent insulin and AMPK signaling pathways, and reduce hepatic glucose output (Munir et al., 2013). In line with these findings, we found that supplementation with N-p-CO could significantly activate PI3K/AKT/GSK3β pathways, upregulate the expression of GLUT2, and improve the hepatocellular glycometabolism in both HG/PA-induced HL-7702 cells and diabetic mice (Figure 8). These findings indicate that the ability of N-p-CO to stimulate hepatocellular glycogenesis production and glucose consumption may be mediated by AKT pathway activation via PI3K phosphorylation, leading to increased p-GSK3β levels and subsequent promotion of glycogen. In other words, the regulation of hepatic glucose metabolism by N-p-CO may be, at least in part, related to the PI3K-dependent activation of AKT. However, the precise mechanism underlying the amelioration of hepatic glucose metabolism dysregulation by N-p-CO through the AKT pathway remains to be further elucidated *in vivo* and *in vitro* utilizing AKT-deficient or CRISPR-Cas9 modified mice models.

## 5 Conclusion

In conclusion, administration with N-p-CO effectively activated a PI3K/AKT/GSK3β signaling pathway and ameliorated oxidative stress in both HG/PA-induced HL-7702 cells and HFD/STZ-induced mice. N-p-CO treatment not only significantly promoted glucose uptake and glycogen synthesis in HG/PA-induced HL-7702 cells, but also exhibited significant hypoglycemic effects by reducing fasting blood glucose and increasing hepatic glycogen production in HFD/STZ-induced mice. These effects demonstrated the significant mitigation of the dysregulation of hepatic glucose metabolism induced by N-p-CO supplementation, and may be attributed to the reduction of the hydroxyl free radical and the activation of a PI3K/AKT/GSK3β signaling pathway. Though more work will be needed to reveal the PI3K/AKT/GSK3β-involved accurate mechanism of action regulated by N-p-CO, the present findings clearly demonstrate that N-p-CO is exploitable as a new candidate for the prevention and treatment of diabetes.

## Data Availability

The raw data supporting the conclusion of this article will be made available by the authors, without undue reservation.

## References

[B1] Adeva-Andany A. Noemi P. F. Carlos F. F. Cristóbal D. G. Cristina P. G. (2016). Liver glucose metabolism in humans. Biosci. Rep. 36, e00416. 10.1042/BSR20160385 27707936 PMC5293555

[B2] Argaev-Frenkel L. Rosenzweig T. (2023). Redox balance in type 2 diabetes: therapeutic potential and the challenge of antioxidant-based therapy. Antioxidants 12, 994. 10.3390/antiox12050994 37237860 PMC10215840

[B3] Ayanlowo A. G. Garádi Z. Boldizsár I. Darcsi A. Nedves A. N. Varjas B. (2020). UHPLC-DPPH method reveals antioxidant tyramine and octopamine derivatives in Celtis occidentalis. J. Pharm. Biomed. Anal. 191, 113612. 10.1016/j.jpba.2020.113612 32980795

[B4] Brasnyó P. Molnár G. A. Mohás M. Markó L. Laczy B. Cseh J. (2011). Resveratrol improves insulin sensitivity, reduces oxidative stress and activates the Akt pathway in type 2 diabetic patients. Br. J. Nutr. 106, 383–389. 10.1017/S0007114511000316 21385509

[B5] Chen J. Cao D. Jiang S. Liu X. Pan W. Cui H. (2022). Triterpenoid saponins from Ilex pubescens promote blood circulation in blood stasis syndrome by regulating sphingolipid metabolism and the PI3K/AKT/eNOS signaling pathway. Phytomedicine 104, 154242. 10.1016/j.phymed.2022.154242 35728385

[B6] Cheng Y. S. Chao J. Chen C. Lv L. L. Han Y. C. Liu B. C. (2019). The PKCβ-p66shc-NADPH oxidase pathway plays a crucial role in diabetic nephropathy. J. Pharm. Pharmacol. 71, 338–347. 10.1111/jphp.13043 30417389

[B7] Dhanya R. Arun K. B. Syama H. P. Nisha P. Sundaresan A. Santhosh Kumar T. R. (2014). Rutin and quercetin enhance glucose uptake in L6 myotubes under oxidative stress induced by tertiary butyl hydrogen peroxide. Food Chem. 158, 546–554. 10.1016/j.foodchem.2014.02.151 24731381

[B8] Ding H. R. Wang J. L. Ren H. Z. Shi X. L. (2018). Lipometabolism and glycometabolism in liver diseases. Biomed. Res. Int. 2018, 1287127. 10.1155/2018/1287127 31205932 PMC6530156

[B9] Ding M. Tang Z. Liu W. Shao T. Yuan P. Chen K. (2021). Burdock fructooligosaccharide attenuates high glucose-induced apoptosis and oxidative stress injury in renal tubular epithelial cells. Front. Pharmacol. 12, 784187. 10.3389/fphar.2021.784187 34955856 PMC8695902

[B10] Elkhalifa A. M. E. Nazar M. Ali S. I. Khursheed I. Taifa S. Ahmad Mir M. (2024). Novel therapeutic agents for management of diabetes mellitus: a hope for drug designing against diabetes mellitus. Life 14, 99. 10.3390/life14010099 38255714 PMC10821096

[B11] Ge Z. Zhang P. Hong T. Tang S. Meng R. Bi Y. (2015). Erythropoietin alleviates hepatic insulin resistance via PPARγ-dependent AKT activation. Sci. Rep. 8, 17878. 10.1038/srep17878 PMC4672330 26643367

[B12] Hao J. Bei J. Li Z. Han M. Ma B. Ma P. (2022). Qing`e Pill inhibits osteoblast ferroptosis via ATM serine/threonine kinase (ATM) and the PI3K/AKT pathway in primary osteoporosis. Front. Pharmacol. 13, 902102. 10.3389/fphar.2022.902102 35865965 PMC9294279

[B13] Hasandeep S. Rajanpreet S. Arshdeep S. Harshbir S. Gurpreet S. Sarabjit K. (2023). Role of oxidative stress in diabetes-induced complications and their management with antioxidants. Arch. Biochem. Biophys. 2023, 2243651. 10.1080/13813455.2023.2243651

[B14] He C. Wang D. Wang R. Huang Y. Huang X. Shen S. (2022). Epigallocatechin gallate induces the demethylation of actinin alpha 4 to inhibit diabetic nephropathy renal fibrosis via the NF-KB signaling pathway *in vitro* . Dose Response 20 (2), 15593258221105704. 10.1177/15593258221105704 35706474 PMC9189538

[B15] Hisashi M. Atsuhi I. Lim C. H. Tamio U. Naotaka F. (1998). Induction of N-p-coumaroyloctopamine in potato tuber disks by 3-1, 3-glucooligosaccharide. J. Pestic. Sci. 23, 49–53. 10.1584/jpestics.23.49

[B16] Hu C. L. Wang C. Y. X Y. Yao X. M. (2020). Dapagliflozin therapy curative effect observation on nonalcoholic fatty liver disease in patients with type 2 diabetes mellitus. Indian J. Pharm. Sci. 82, 122–129. 10.36468/pharmaceutical-sciences.spl.155

[B17] Hu C. M. Liang B. W. Sun J. P. Li J. Y. Xiong Z. Wang S. H. (2024). Synthesis and biological evaluation of indole derivatives containing thiazolidine-2,4-dione as α-glucosidase inhibitors with antidiabetic activity. Eur. J. Med. Chem. 264, 115957. 10.1016/j.ejmech.2023.115957 38029465

[B18] Huang Q. Chen L. Teng H. Song H. B. Wu X. Q. Xu M. Y. (2015). Phenolic compounds ameliorate the glucose uptake in HepG2 cells' insulin resistance via activating AMPK: anti-diabetic effect of phenolic compounds in HepG2 cells. J. Funct. 19, 487–494. 10.1016/j.jff.2015.09.020

[B19] Huang T. S. Wu T. Wu Y. D. Li X. H. Tan J. Shen C. H. (2023). Long-term statins administration exacerbates diabetic nephropathy via ectopic fat deposition in diabetic mice. Nat. Commun. 14, 390. 10.1038/s41467-023-35944-z 36693830 PMC9873739

[B20] Jeppe S. S. Nils T N. Dan S. (2014). Assessment of constituents in Allium by multivariate data analysis, high-resolution α-glucosidase inhibition assay and HPLC-SPE-NMR. Food Chem. 161, 192–198. 10.1016/j.foodchem.2014.03.062 24837940

[B21] Jha D. Mazumder P. M. (2019). High fat diet administration leads to the mitochondrial dysfunction and selectively alters the expression of class 1 GLUT protein in mice. Mol. Biol. Rep. 46, 1727–1736. 10.1007/s11033-019-04623-y 30725350

[B22] Jiang P. Ren L. J. Zhi L. Yu Z. Lv F. Xu F. (2021). Negative regulation of AMPK signaling by high glucose via E3 ubiquitin ligase MG53. Mol. Cell 81, 629–637.e5. 10.1016/j.molcel.2020.12.008 33400924

[B23] Jiang Y. X. Li W. Wang J. Wang G. G. (2020). Cardiac dysfunction is attenuated by ginkgolide B via reducing oxidative stress and fibrosis in diabetic rats. Iran. J. Basic Med. Sci. 23, 1078–1084. 10.22038/ijbms.2020.44210.10358 32952955 PMC7478264

[B24] Kane N. S. Hoogendoorn C. J. Tanenbaum M. L. Gonzalez J. S. (2018). Physical symptom complaints, cognitive emotion regulation strategies, self-compassion and diabetes distress among adults with Type 2 diabetes. Diabet. Med. 35, 1671–1677. 10.1111/dme.13830 30264898 PMC7313242

[B25] Krawczyk M. Burzynska-Pedziwiatr I. Wozniak L. A. Bukowiecka-Matusiak M. (2023). Impact of polyphenols on inflammatory and oxidative stress factors in diabetes mellitus: nutritional antioxidants and their application in improving antidiabetic therapy. Biomolecules 13, 1402. 10.3390/biom13091402 37759802 PMC10526737

[B26] Kukavica B. Škondrić S. Trifković T. Mišić D. Gašić U. Topalić-Trivunović L. (2024). Comparative polyphenolic profiling of five ethnomedicinal plants and their applicative potential in the treatment of type 2 diabetes. J. Ethnopharmacol. 320, 117377. 10.1016/j.jep.2023.117377 37939910

[B27] Kumar S. Behl T. Sachdeva M. Sehgal A. Kumari S. Kumar A. (2021). Implicating the effect of ketogenic diet as a preventive measure to obesity and diabetes mellitus. Life Sci. 264, 118661. 10.1016/j.lfs.2020.118661 33121986

[B28] Liu W. B. Wang H. L. Chen L. Tang B. Ke G. Wang S. (2022). Cucurbitacin E inhibits cellular proliferation and induces apoptosis in melanoma by suppressing HSDL2 expression. Chin. Med. 17 (1), 28. 10.1186/s13020-022-00582-y 35193614 PMC8862504

[B29] Ma M. Y. Wei N. Yang J. R. Ding T. T. Song A. P. Chen L. R. (2023). Schisandrin B promotes senescence of activated hepatic stellate cell via NCOA4-mediated ferritinophagy. Pharm. Biol. 61, 621–629. 10.1080/13880209.2023.2189908 37010139 PMC10071970

[B30] Maria P. Mattias C. (2017). Adenosine signaling in diabetes mellitus and associated cardiovascular and renal complications. Mol. Asp. Med. 55, 62–74. 10.1016/j.mam.2016.12.001 28089906

[B31] Min X. F. Guo S. Q. Lu Y. J. Xu X. T. (2024). Investigation on the inhibition mechanism and binding behavior of cryptolepine to α-glucosidase and its hypoglycemic activity by multi-spectroscopic method. J. Lumin 269, 120437. 10.1016/j.jlumin.2024.120437

[B32] Munir K. M. Chandrasekaran S. Gao F. Quon M. J. (2013). Mechanisms for food polyphenols to ameliorate insulin resistance and endothelial dysfunction: therapeutic implications for diabetes and its cardiovascular complications. Am. J. Physiol. Endocrinol. Metab. 305, E679–E686. 10.1152/ajpendo.00377.2013 23900418 PMC4073986

[B33] Nchiozem-Ngnitedem V. A. Omosa L. K. Bedane K. G. Derese S. Brieger L. Strohmann C. (2020). Anti-inflammatory steroidal sapogenins and a conjugated chalcone-stilbene from Dracaena usambarensis Engl. Fitoterapia 146, 104717. 10.1016/j.fitote.2020.104717 32877711

[B34] Petersen M. C. Vatner D. F. Shulman G. I. (2017). Regulation of hepatic glucose metabolism in health and disease. Nat. Rev. Endocrinol. 13, 572–587. 10.1038/nrendo.2017.80 28731034 PMC5777172

[B35] Qi X. Zheng S. Ma M. Lian N. Wang H. Chen L. (2022). Curcumol Suppresses CCF-mediated hepatocyte senescence through blocking LC3B-Lamin B1 interaction in alcoholic fatty liver disease. Front. Pharmacol. 13, 912825. 10.3389/fphar.2022.912825 35837283 PMC9273900

[B36] Rathinam A. Pari L. (2016). Myrtenal ameliorates hyperglycemia by enhancing GLUT2 through Akt in the skeletal muscle and liver of diabetic rats. Chem. Biol. Interact. 256, 161–166. 10.1016/j.cbi.2016.07.009 27417257

[B37] Rines A. K. Sharabi K. Tavares C. D. Puigserver P. (2016). Targeting hepatic glucose metabolism in the treatment of type 2 diabetes. Nat. Rev. Drug Discov. 15, 786–804. 10.1038/nrd.2016.151 27516169 PMC5751421

[B38] Satman I. Bayirlioglu S. Okumus F. Erturk N. Yemenici M. Cinemre S. (2023). Estimates and forecasts on the burden of prediabetes and diabetes in adult and elderly population in Turkiye. Eur. J. Epidemiol. 38, 313–323. 10.1007/s10654-022-00960-8 36696072 PMC9875179

[B39] Shah M. A. Haris M. Faheem H. I. Hamid A. Yousaf R. Rasul A. (2022). Cross-talk between obesity and diabetes: introducing polyphenols as an effective phytomedicine to combat the dual sword diabesity. Curr. Pharm. Des. 28, 1523–1542. 10.2174/1381612828666220628123224 35762558

[B40] Song J. Xu H. F. Zhang W. Yang C. Y. Li L. Luan J. J. (2022). Impact of solute carrier family 47 member 1 gene polymorphism detection on therapeutic effect of diabetes. Int. J. Pharmacol. 18, 398–406. 10.3923/ijp.2022.398.406

[B41] Sourbh S. G. Jeena G. (2022). Polyol pathway and redox balance in diabetes. Pharmacol. Res. 182, 106326. 10.1016/j.phrs.2022.106326 35752357

[B42] Sun S. Li S. Du Y. Wu C. Zhang M. Li J. (2020a). Anti-inflammatory effects of the root, stem and leaf extracts of Chloranthus serratus on adjuvant-induced arthritis in rats. Pharm. Biol. 58, 528–537. 10.1080/13880209.2020.1767159 32503379 PMC8641675

[B43] Sun S. Wang Y. Du Y. Sun Q. He L. Zhu E. (2020b). Oxidative stress-mediated hepatotoxicity in rats induced by ethanol extracts of different parts of Chloranthus serratus. Pharm. Biol. 58, 1277–1289. 10.1080/13880209.2020.1859552 33355514 PMC7759245

[B44] Sun S. Zhang J. Li H. Du Y. Li S. Li A. (2021). Anti-inflammatory activity of the water extract of Chloranthus serratus roots in LPS-stimulated RAW264.7 cells mediated by the Nrf2/HO-1, MAPK and NF-κB signaling pathways. J. Ethnopharmacol. 271, 113880. 10.1016/j.jep.2021.113880 33508367

[B45] Sun X. Zheng Y. Q. Tian Y. Q. Xu Q. X. Liu S. C. Li H. H. (2023a). Astragalus polysaccharide alleviates alcoholic-induced hepatic fibrosis by inhibiting polymerase I and transcript release factor and the TLR4/JNK/NF-κB/MyD88 pathway. J. Ethnopharmacol. 314, 116662. 10.1016/j.jep.2023.116662 37207880

[B46] Sun Y. Guo L. Q. Wang D. G. Xing Y. J. Bai Y. P. Zhang T. (2023b). Metformin alleviates glucolipotoxicity-induced pancreatic β cell ferroptosis through regulation of the GPX4/ACSL4 axis. Eur. J. Pharmacol. 956, 175967. 10.1016/j.ejphar.2023.175967 37549729

[B47] Taniguchi C. M. Kondo T. Sajan M. Luo J. Bronson R. Asano T. (2006). Divergent regulation of hepatic glucose and lipid metabolism by phosphoinositide 3-kinase via Akt and PKClambda/zeta. Cell Metab. 3, 343–353. 10.1016/j.cmet.2006.04.005 16679292

[B48] Tao Z. S. Zhou W. S. Xu H. G. Yang M. (2020). Simvastatin can enhance the osseointegration of titanium rods in ovariectomized rats maintenance treatment with valproic acid. Biomed. Pharmacother. 132, 110745. 10.1016/j.biopha.2020.110745 33068938

[B49] Volpe C. M. O. Villar-Delfino P. H. Dos Anjos P. M. F. Nogueira-Machado J. A. (2018). Cellular death, reactive oxygen species (ROS) and diabetic complications. Cell Death Dis. 9, 119. 10.1038/s41419-017-0135-z 29371661 PMC5833737

[B50] Wang L. Wang P. Wang D. Tao M. Xu W. Olatunji O. J. (2020). Anti-inflammatory activities of kukoamine A from the root bark of lycium chinense Miller. Nat. Prod. Commun. 15, 1934578X2091208. 10.1177/1934578X20912088

[B51] Wang R. Mu J. (2021). Arbutin attenuates ethanol-induced acute hepatic injury by the modulation of oxidative stress and Nrf-2/HO-1 signaling pathway. J. Biochem. Mol. Toxicol. 35, e22872. 10.1002/jbt.22872 34346143

[B52] Wang S. Chen X. Cheng J. Cai T. Wu X. Cheng Z. (2021). Calunduloside E inhibits HepG2 cell proliferation and migration via p38/JNK-HMGB1 signalling axis. J. Pharmacol. Sci. 147, 18–26. 10.1016/j.jphs.2021.05.005 34294368

[B53] Wu X. Z. Zhu W. J. Lu L. Hu C. M. Zheng Y. Y. Zhang X. (2023). Synthesis and anti-α-glucosidase activity evaluation of betulinic acid derivatives. Arab. J. Chem. 16, 104659. 10.1016/j.arabjc.2023.104659

[B54] Xing Y. J. Liu B. H. Wan S. J. Cheng Y. Zhou S. M. Sun Y. (2021). A SGLT2 inhibitor dapagliflozin alleviates diabetic cardiomyopathy by suppressing high glucose-induced oxidative stress *in vivo* and *in vitro* . Front. Pharmacol. 12, 708177. 10.3389/fphar.2021.708177 34322029 PMC8311522

[B55] Xu S. He L. Ding K. Zhang L. Xu X. Wang S. (2020). Tanshinone IIA ameliorates streptozotocin-induced diabetic nephropathy, partly by attenuating PERK pathway-induced fibrosis. Drug Des. Devel Ther. 14, 5773–5782. 10.2147/DDDT.S257734 PMC7780857 33408464

[B56] Yan Z. Cao X. J. Sun S. X. Sun B. Gao J. (2023). Inhibition of GSK3B phosphorylation improves glucose and lipid metabolism disorder. Biochim. Biophys. Acta Mol. Basis Dis. 1869, 166726. 10.1016/j.bbadis.2023.166726 37146915

[B57] Ye X. Chen W. Huang X. F. Yan F. J. Deng S. G. Zheng X. D. (2024). Anti-diabetic effect of anthocyanin cyanidin-3-O-glucoside: data from insulin resistant hepatocyte and diabetic mouse. Nutr. Diabetes 14, 7. 10.1038/s41387-024-00265-7 38429305 PMC10907696

[B58] Ye X. Chen W. Tu P. Jia R. Liu Y. Q. T. Tang Q. (2022). Antihyperglycemic effect of an anthocyanin, cyanidin-3-O-glucoside, is achieved by regulating GLUT-1 via the Wnt/β-catenin-WISP1 signaling pathway. Food Funct. 13, 4612–4623. 10.1039/d1fo03730g 35357376

[B59] Yoshikawa T. Mifune Y. Inui A. Nishimoto H. Yamaura K. Mukohara S. (2022). Influence of diabetes-induced glycation and oxidative stress on the human rotator cuff. Antioxidants 11, 743. 10.3390/antiox11040743 35453426 PMC9032678

[B60] Zhang L. Ren X. H. He J. Li W. N. Hao J. W. Jing Y. R. (2021). Protective effect of Phaeoporus obliquus polysaccharide against acute liver injury induced by carbon tetrachloride and alcohol in mice. Pak J. Pharm. Sci. 34, 649–656. 10.36721/PJPS.2021.34.2.REG.649-656.1 34275842

[B61] Zhang S. P. Zhu P. Yuan J. N. Cheng K. M. Xu Q. X. Chen W. (2023). Non-alcoholic fatty liver disease combined with rheumatoid arthritis exacerbates liver fibrosis by stimulating co-localization of PTRF and TLR4 in rats. Front. Pharmacol. 14, 1149665. 10.3389/fphar.2023.1149665 37346294 PMC10279862

[B62] Zhang X. S. Lu Y. Li W. Tao T. Peng L. Wang W. H. (2021). Astaxanthin ameliorates oxidative stress and neuronal apoptosis via SIRT1/NRF2/Prx2/ASK1/p38 after traumatic brain injury in mice. Brit J. Pharmacol. 178, 1114–1132. 10.1111/bph.15346 33326114

[B63] Zhou Y. Liu L. Xiang R. Bu X. Qin G. Z. Dai J. J. (2023). Arctigenin mitigates insulin resistance by modulating the IRS2/GLUT4 pathway via TLR4 in type 2 diabetes mellitus mice. Int. Immunopharmacol. 114, 109529. 10.1016/j.intimp.2022.109529 36481528

